# Analysis of Human Body Shadowing Effect on Wireless Sensor Networks Operating in the 2.4 GHz Band

**DOI:** 10.3390/s18103412

**Published:** 2018-10-11

**Authors:** Łukasz Januszkiewicz

**Affiliations:** Institute of Electronics, Lodz University of Technology, ul. Wólczańska 211/215, 90-924 Łódź, Poland; lukasz.januszkiewicz@p.lodz.pl; Tel.: +48-691-565-277

**Keywords:** wireless sensor networks, body shadowing, path loss, computational electromagnetics, FDTD, UTD

## Abstract

Miniaturized wireless sensors are designed to run on limited power resources, requiring minimization of transmit power and lowering of the fade margin in the link budget. One factor that has an important impact on wireless sensor network design is path loss between the transmitter and the receiver. This paper presents an analysis of the influence of human bodies on path loss in the 2.4 GHz band, which is commonly used for wireless sensor networks. The effect of body shadowing was first analyzed in full wave computer simulations using the finite-difference time-domain method. Due to the high numerical burden, the simulations were limited to only a small region around the human body. To analyze the performance of networks in larger indoor environments, a human body model is proposed that can be used for simulations with a ray-based computer program. The proposed model of human body is the main contribution of this paper. It was used to analyze the body shadowing effect in a typical indoor environment. The results were found to be in good agreement with measurements.

## 1. Introduction

Wireless sensor networks have been the subject of intensive research in recent years. The development of highly miniaturized electronic integrated circuits has made possible the design of modern sensors that can perform very complex functions, such as measuring the physical parameters of an environment or the physiological parameters of people, and transmit this measurement data via a wireless link to the receiver. Wireless sensor networks have found applications in many fields, including agriculture [1], marine environment monitoring [2], ambient assisted living [3], industry [4], and healthcare [5]. Wireless sensor networks often operate without a dedicated licensed frequency band, utilizing open bands in the existing radio environment (e.g., 2.4 GHz Industrial, Scientific, Medical—ISM) that do not require licenses [6]. Because the transmit power of open bands is limited, they are suitable when the operating range of a single node is not very large (usually tens to hundreds of meters). On the other hand, whole networks composed of numerous nodes can cover an entire building or even a group of buildings [7]. Use of the 2.4 GHz band for wireless sensor networks is greatly facilitated by the large numbers of miniature transceivers available. There are also many transmission protocols suitable for wireless sensor networks, such as IEEE 802.15.4, Bluetooth, and ANT [8].

The capacity of miniature batteries still places an important limitation on the performance of wireless sensor networks with very small nodes [9,10]. This limitation also affects the performance of the wireless links that are used to transmit data from the sensor nodes. Due to the constraints on the available power resources, designers need to minimize the transmit power and lower the fading margin in the wireless link power budget. One of the important factors to consider in wireless sensor network design is the path loss that occurs, depending on the physical environment, between the transmitter and the receiver. In high frequency bands (with frequency measured in GHz), path loss can be increased by the presence of additional obstacles in the transmission path, in particular by people in proximity to the transmitter. This effect is called body shadowing, and can have an important impact on the performance of wireless systems. 

**Figure 1:**
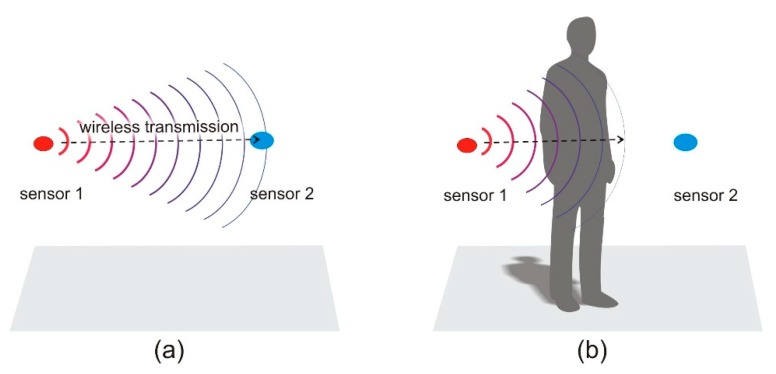
Influence of body shadowing on wireless transmission between sensors: (**a**) No shadowing, successful transmission; (**b**) body shadowing that affects transmission

Figure 1 shows two simplified scenarios in which wireless transmission between sensors occurs with and without human body shadowing. In the first case (Figure 1a), there is no obstacle between the transmitting node 1 and receiving node 2. Assuming signal attenuation in an open environment there will be successful transmission. In the second case (Figure 1b), the presence of a human body in the transmission path between the nodes causes additional attenuation of the wireless link and body shadowing occurs. If the wireless sensor has a low power transmitter and low fading margin, this may lead to a loss of packets or even break the wireless link. Therefore, it is important for designers of wireless sensor networks to consider the additional path loss introduced by human bodies.

The body-shadowing effect has been investigated by researchers working on wireless systems as well as on body area networks in a very wide range of frequencies [11,12,13,14,15,16,17,18,19,20,21,22,23,24]. Statistical analysis is often used to model this effect, including the time variation caused by people in motion and the properties of the indoor environment, which can influence the propagation of the signal [13,14,15]. Other research has applied the experimental approach, taking numerous measurements of path loss due to human bodies in typical propagation environments [16,17,18]. Based on the results, propagation models have then been constructed with specific parameters that can be modified for different operating scenarios [19,20]. 

The 2.4 GHz band is widely used for wireless transmission systems because in most countries, this is an unlicensed band. For this frequency, the study of the body shadowing effect was presented in the literature [14,21,22,23,24]. In [21], a statistical approach was applied for the analysis and modeling of the impact of path loss on the wireless link that is operating in the 2.4 GHz band. Also in [22], a statistical signal reception model for shadowed body-centric communications was proposed. The parameters of channel models proposed in the literature are extracted from channel measurements and can be used in statistical analysis of wireless system performance [23,24].

This paper presents an analysis using computer modeling of the effect of body shadowing on wireless link performance in the very popular 2.4 GHz band. The effect of body shadowing was first analyzed in full wave computer simulations using the finite-difference time-domain (FDTD) method. Due to the high numerical burden of this method, the analysis was limited to only a small region around the human body. To analyze the performance of wireless sensor networks across larger distances, including indoor propagation environments, a simplified human body model is proposed that can be used in simulations made with a ray-based computer program utilizing the unified theory of diffraction (UTD). A similar model of the human body was first proposed by the author for simulations of the human body shadowing effect in 5 G wireless systems operating in the 3.4 GHz band [25]. The model required reconfiguration for simulations in the 2.4 GHz band, which is particularly useful for wireless sensor networks. The results of the simulations were found to be in good agreement with measurements, suggesting that the model is suitable for analyzing wireless sensor networks operating across large areas, including in buildings with obstacles. 

The main contribution of this paper is the proposed human body model dedicated for a ray-based computer program that can be successfully used for body shadowing simulations in the 2.4 GHz band. The proposed model has practical value because the design of wireless systems that operate in the indoor environment is often performed with a ray-based software that is computationally effective in the modelling of large objects. In this kind of simulation software (in contrast to full-wave simulators), there were no human body models that could be used to model the shadowing effect. 

## 2. Materials and Methods

### 2.1. Analysis of Shadowing Effect with Finite-Difference Time-Domain Based Program

The human body shadowing effect was analyzed using a computer program based on the FDTD numerical method. This method is commonly used for computer simulations in high frequency electromagnetics. Thanks to the direct implementation of Maxwell equations formulated in the time domain, it is particularly useful for the analysis of objects with very complex structures, made from various materials (dielectrics and conductors) [26,27]. The simulations of the body shadowing effect presented in this paper were performed using Remcom XFdtd™ software ver. 7.6.0.2 [28], which employs the FDTD method. This software can be used for the simulation of diverse objects segmented into cubic volumetric elements (voxels), following the procedure suggested by Yee [29]. 

The finite-difference time-domain method is recommended for studying the influence of electromagnetic waves on the human body [30]. Data on the electromagnetic properties of human body tissues are now available for a wide range of frequencies [31]. Many numerical models of the human body have been developed for the purposes of computer simulations employing the FDTD method. These differ mainly in the number of tissues modeled and in the anatomical accuracy of the body structure. The research presented here was performed using the NMR Hershey model, which is available in XFdtd™ software ver. 7.6.0.2 [28]. This anthropomorphic heterogeneous model of the human body can be deployed with different resolutions, from 1 mm to 10 mm.

Software that uses the FDTD method performs a segmentation of objects into voxels. The voxel size corresponds to the length of the electromagnetic wave that penetrates the object under analysis. The dimensions of the voxels into which the object is divided influences the accuracy of the calculations. A commonly applied rule is to assume the length of the side of the cell to be equal to one tenth of the length of the shortest electromagnetic wave in the simulated object *λ*_min_ (1):

(1) $$\lambda_{\min}=\frac{c_{0}}{f_{c}\cdot \sqrt{\mu_{r}\varepsilon_{r}}}\;$$

where: *λ*_min_—the length of the shortest wave in the model; *c*_0_—the velocity of light in a vacuum; *f_c_*—the wave frequency; *µ_r_*—the maximum relative magnetic permeability of the model object; and *ε_r_*—the maximum relative electric permittivity of the modeled object.

The need to model the entire simulation domain using voxels with sides a faction of a wavelength limits the use of this method to problems where the physical size is comparable to tens or maximally hundreds of the wavelength. There must be sufficient computer memory to store the data describing material properties and electromagnetic field parameters for all the voxels that fill the simulation domain. In simulations that include human body models, due to the shortening of the wave that occurs as it passes through dielectric material, the voxel size is smaller than the wavelength in free space. In this study, for a human body with average properties, the relative electric permittivity of the model material was *ε* = 38.5, while its specific conductance was *σ* = 2.4 S/m. These values are typical for models used in tests of cellular system terminals [32] and can be used to approximate the average wavelength of a human body. For the 2.45 GHz frequency, the average wavelength in the human body according to formula 1 is *λ*_min_ = 19.7 mm. The average voxel size should then be 1.97 mm. In Remcom XFdtd™, the closest approximation of this size is a human body model with 2 mm voxels. This was used for further simulations. 

**Figure 2:**
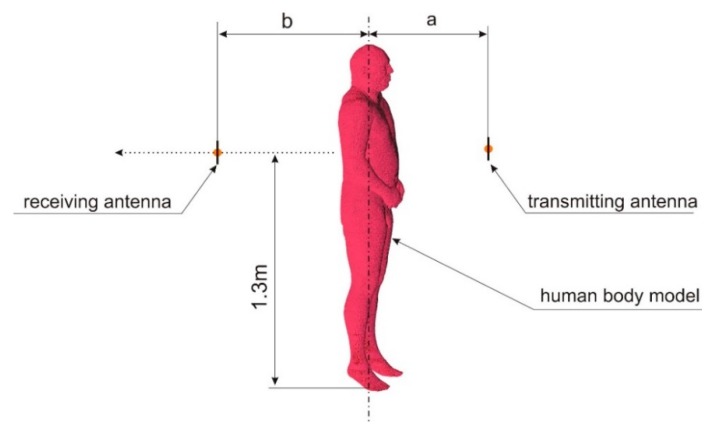
Geometry of the numerical experiment performed in XFdtd™ software.

To simulate the body shadowing effect in Remcom XFdtd™ software, coupling between two antennas was simulated. Simulations were performed for the two cases, presented in Figure 1. In the first case (presented in Figure 1a), it was assumed that there were no obstacles between the antennas while in the second case (Figure 1b), a human body was placed between the antennas. The detailed geometry of the numerical experiment is presented in Figure 2. The antennas were placed 1.3 m above the feet of the model body. The transmitting antenna was placed at distance *a* from the body axis. The receiving antenna was located at distance *b*. Half-wave dipoles were used, shortened for resonance at 2.45 GHz. The absorbing boundary condition was applied to model the location of the antenna and the human body in free space, without any obstacles or reflecting planes that might have influenced the path loss between the antennas. The absorbing boundary condition that limits the simulation domain in the vertical direction touched the feet of the model body and was placed 40 cm above its head. This models the case of a person standing on the floor, but in this case, only the body and the distance between the antennas have any influence on path loss. No diffraction from the soles of the feet or reflections from the floor are included in the model.

**Figure 3:**
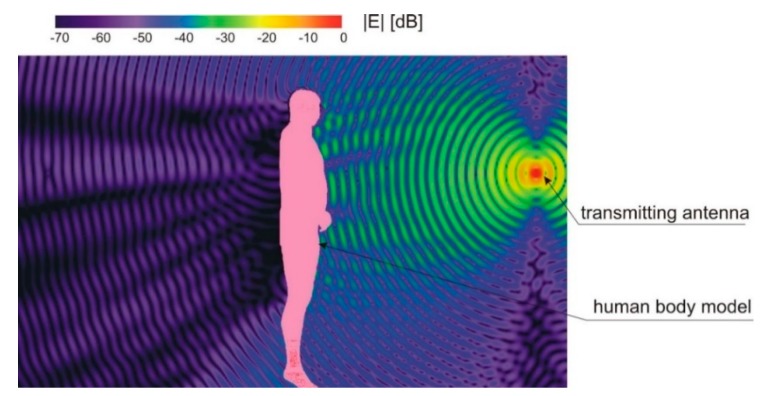
Electric field intensity in proximity to a human body simulated in XFdtd.

The first analysis of the body shadowing effect simulated the electric field distribution in the area behind the human body. The results of the simulations of electric field intensity generated by the transmitting antenna placed at a distance, *a* = 1.5 m, are presented in Figure 3. As can be seen, in the shadow region on the opposite side of the body to the transmitting antenna, the electric field intensity is significantly lower than on the side of the transmitting antenna. 

Another important factor influencing electric field intensity in the shadow region is the distance of the transmitting antenna from the body. Assuming that the transmitting antenna is placed in front of the body and the receiving antenna is behind, the shadowing effect will influence the path loss between the antennas. To analyze this effect, a series of simulations was performed for different distances between the body and the receiving antenna. The path loss between the two antennas was simulated in the XFdtd™ program with the transmitting antenna in three fixed positions (parameter *a* in Figure 2), with the receiving antenna at different distances (parameter *b* in Figure 2). The 3 fixed positions for the transmitting antenna (parameter *a*) were 1 m, 2 m, and 3 m from the axis of human body model. As the reference case, the free space path loss was simulated with no human body model between the antennas and parameter *a* equal to 2 m. 

**Figure 4:**
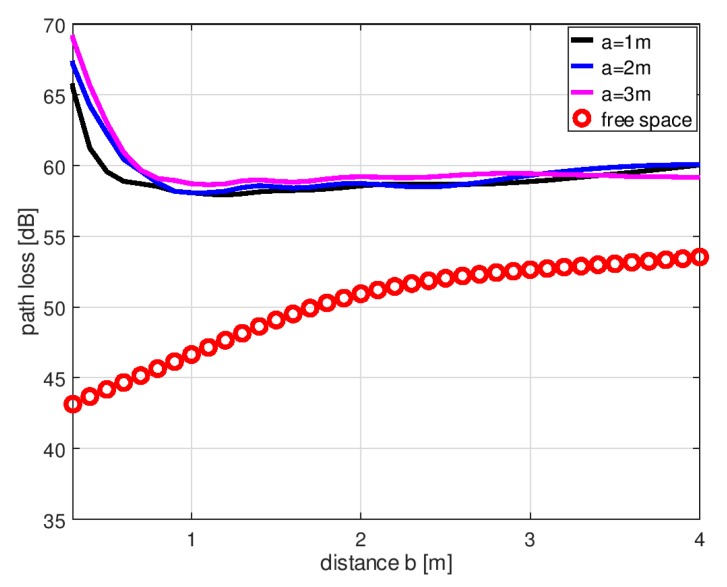
Path loss between transmitting and receiving dipoles simulated in XFdtd™ program as a function of receiving antenna distance *b* defined in Figure 2. Results for free space (no human body model and parameter *a* equal to 2 m) and for the shadowed case with different transmitting antenna spacings (*a* = 1, 2, 3 m).

The results of the body shadowing simulations are presented in Figure 4. The body shadowing effect was the most intense when the receiving antenna was in close proximity to the human body (0.3–0.4 m from the axis of the body). Free space path loss was approximately 44 dB while in the shadowed case, it increased to approximately 65 dB (depending on the position of the transmitting antenna). With the receiving antenna at a distance of 1 m from the body, the difference between the free space and the shadowed case was still high (approx. 10 dB). At larger distances, the differences reduced, reaching approx. 6 dB at 4 m. These results show the influence that the presence of a human body can have on the operation of a wireless sensor network. When a human body is close to the wireless sensor, the path loss may increase (from 10 dB to 20 dB) and could exceed the fading margin of the link. 

Little difference was noted between the path loss simulated for transmitting antennas located at distances of 2 m and 3 m from the axis of the body. Only with the transmitting antenna located at a distance of *a* = 1 m was the path loss slightly lower in proximity to the human body. For further analysis, the distance, *a* = 2 m, was selected, because in indoor environments, larger distances between transmitting sensors and human bodies are less likely. 

The analysis of the body shadowing effect using the FDTD method in this study was limited to only the regions in closest proximity to the human body. Due to the high requirements of the simulation for computer memory, it was possible only to model the human body and two antennas at a maximum distance of 7 m from each other (the maximum values for parameters *a* and *b* were 3 m and 4 m, respectively). In XFdtd™, it is possible to adjust the voxel size to the length of the wave in a particular object, but even with this possibility, the requirements for computer memory were high. The voxel sizes used in this study were 2 mm for the body model and 10 mm for the air volume. The two half-wave dipole antennas used to identify the path loss in the numerical experiment were modeled using 0.5 mm voxels suitable for thin wires. The computer memory required to model the entire geometry of the experiment was 9.8 GB. The simulations were run on a computer equipped with two Nvidia Tesla 2070 GPU cards with multi-core (448 cores) processors capable of running massively parallel computations and with 6 GB of memory each. With this setup, for each position of the receiving antenna, the simulation time lasted from 20 min up to 85 min. This shows the limitation of FDTD-based software for simulating the body shadowing effect on wireless sensor networks inside buildings. To simulate such networks in modern buildings, it is necessary to model not only much larger volumes, but also the walls and other obstacles. For this, FDTD-based programs require far more than 12 GB of computer memory. The time needed for the simulations would also be impractically high, especially for wireless sensor network designs demanding numerous simulations. These limitations motivated the author to develop a numerical model of the human body applicable in the numerically effective method that can be used for large scale simulations of indoor wireless sensor networks, including the body shadowing effect.

### 2.2. Human Body Model for Shadowing Analysis in UTD-Based Program

Simulations of the body shadowing effect based on the FDTD method are of limited use for the design of wireless sensor systems. The computer resources required constrain the physical size of the geometry that can surround the body model in simulations. For the 2.4 GHz frequency of interest in this study, it was possible to simulate path loss only over a distance of a few meters from the human body. This is far too small a region to model the path loss in indoor wireless sensor networks. Ray-based numerical methods are more suitable for this task [33,34]. They can simulate the interactions of large objects even with very short waves, utilizing the resources of a typical personal computer. This motivated the author to elaborate a model of the human body that could be used for simulating the shadowing effect in software that uses ray-based methods for wireless system design.

The next stages of the research utilized the Remcom XGtd™ program ver. 3.1.2.0 [35]. This is a general purpose electromagnetic simulation program based on the analysis of rays radiating from the source and interacting with simulated objects. The program can be used to model the propagation of electromagnetic waves in the vicinity of objects that are large in comparison to the wavelength (aircraft, vehicles, anechoic chambers, etc.). It is particularly useful for studying cases where antennas are positioned in large objects, such as vehicles or buildings. This motivated the author to use XGtd™ to simulate a wireless sensor network operating inside a building. The XGtd™ program models the physical characteristics of objects and anechoic chambers, performs the electromagnetic calculations, and then evaluates the signal propagation characteristics. It can be used to predict how the transmitters and receivers will interact with large objects, calculating the signal strength, path loss, and far zone antenna gain.

Simulations in XGtd™ are made by shooting rays from the transmitters and propagating them through the defined geometry of the modeled object. The rays interact with the geometrical features of the objects as they make their way to the receiver. Ray interactions that are simulated include reflections from feature faces, diffractions around feature edges, and transmission through feature faces. The ray-based solvers use the uniform theory of diffraction (UTD) to evaluate the electric field along the ray path [36,37,38]. Accurate results are provided when the simulated object is significantly large compared to the wavelength of the propagating wave. This requirement is only partially fulfilled in the studied case of human body shadowing in the 2.4 GHz band because the wavelength is equal to 0.125 m and the body height is 1.8 m. However, the UTD method has been shown in the literature to be adequate for modeling the body shadowing effect in the 2.4 GHz band [13], albeit not taking fully into account the shape of a human body. In [25], a simplified human body model was used to simulate body shadowing in the 3.6 GHz band, demonstrating the possible applicability of the UTD method to the considered case. For typical applications, UTD-based models provide accurate predictions from approximately 100 MHz to approximately 100 GHz and the considered band is within this range. The three-dimensional propagation model applied in XGtd™ at each receiver location combines and evaluates the contributions from arriving ray paths to determine predicted quantities, such as electric and magnetic field strength, received power, interference measures, path loss, delay spread, direction of arrival, impulse response, electric field vs. time, electric field vs. frequency, and the power delay profile. In the case of a model that consists of transmitting and receiving antennas, the path loss is also calculated. 

Modeling the body shadowing effect using a ray-based program requires simplification of the human body shape. In [13], the authors considered different elementary objects, such as a rectangular blade, a parallelepiped, or a circular cylinder, for modeling the human body. With such a simplistic approach, it was found that it was possible to estimate only the mean attenuation introduced by the body, averaged over different positions of a person relative to the transmitting antenna. It was not possible to identify the shadow region in the proximity of the human body, which is important for designers of wireless sensor networks utilizing wireless nodes located close to people in buildings.

Cylinder-based models of the human body presented in the literature [39,40,41] show that this geometrical figure is adequate for both simulations and measurements of the interactions between a human body and electromagnetic waves. In previous work by the author [25], a dielectric cylinder-based model of the human body was successfully applied in the XGtd™ program to simulate the body-shadowing effect in the 3.6 GHz band. It was necessary to further simplify the original circular cylinders by using polygonal prisms. The number of side walls used to approximate the body cylinder was found to be frequency dependent and had a major influence on the results of body shadowing simulations elaborated in the XGtd™ program. 

**Figure 5:**
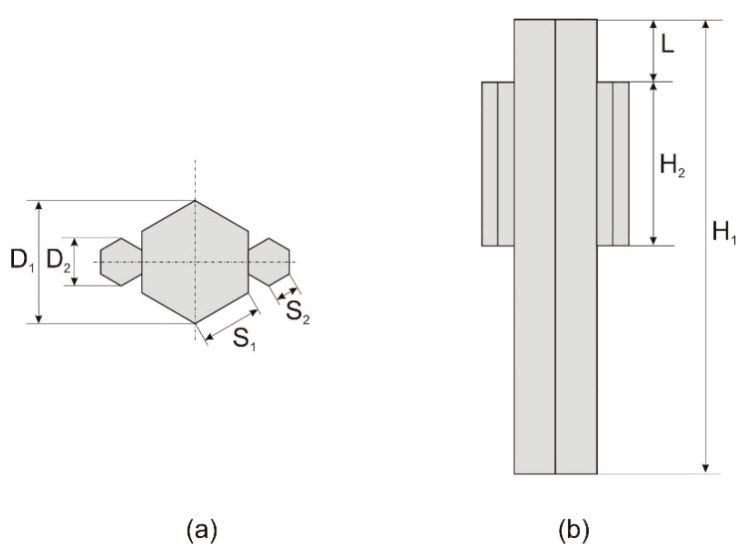
Geometry of the simplified human body model in the XGtd™ program: (**a**) Top view; (**b**) Front view.

The cylindrical model of the human body approximated with polygonal prisms developed and applied previously by the author to study the body-shadowing effect in the 3.6 GHz band was used as the basis for designing a body model suitable for simulating body shadowing in XGtd for the 2.4 GHz band. The number of sidewalls used for cylinder approximation was optimized to obtain the best correspondence between the body shadowing simulations and the results of FDTD. The human body model was simplified using 2 polygonal prisms, as shown in Figure 5. The dimensions were as follows: *D*_1_ = 310 mm, *D*_2_ = 110 mm, *H*_1_ = 1800 mm, *H*_2_ = 530 mm, *L* = 200 mm. The material used to model the human body had the same properties as the human body model proposed by the author to study the body-shadowing effect in the 3.6 GHz band [25]. Its relative electric permittivity was *ε* = 52, and its specific conductance was *σ* = 1.8 S/m. Those values of material parameters were also successfully used by the author for simplified numerical models of the human body in the FDTD method [40,41]. The simulations were performed in a free space environment, assuming that the only obstacle between the dipole antennas was the human body model. 

**Figure 6:**
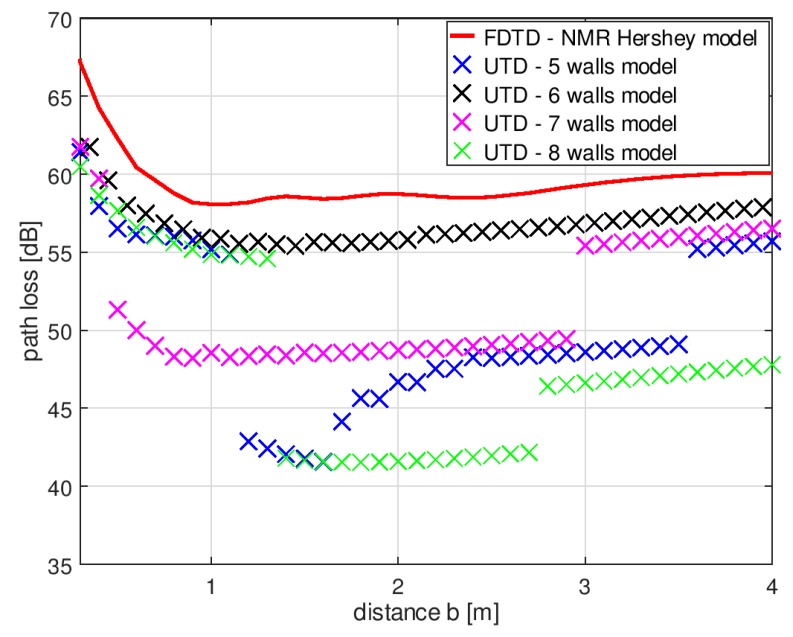
Path loss simulated in the XGtd™ program for different numbers of prism sidewalls compared to path loss simulated in FDTD based program.

Figure 6 presents the results of path loss simulations in XGtd™ with different numbers of prism sidewalls used to approximate the main cylinder. The influence of the number of sidewalls used to model the arm cylinders was negligible. In further analysis, only the number of sidewalls in the main prism was considered, while the number of sidewalls approximating the arms was adjusted to match the number of walls in the main prism. The results were compared to the path loss simulated in XFdtd. The distance of the transmitting dipole from the body axis (*a*) was assumed to be equal to 2 m. The reference result obtained in XFdtd™ was calculated for the same spatial configuration. The differences between the path losses obtained with XGtd™ and XFdtd™ depended on both (1) the number of sidewalls approximating the main body cylinder, and (2) the distance (*b*) between the receiving antenna and the human body. To find the best model configuration, the median values for the path loss across three distance ranges were compared. 

Table 1 presents the differences between the median values for path loss obtained with the FDTD and UTD methods. These results were obtained for different numbers of sidewalls approximating the main body cylinder and with three distance ranges: 0.5–1 m, 1–2 m, and 2–4 m. The smallest differences between the methods were obtained for the model with six wall prisms. For the first distance range, in which the body shadowing effect had the strongest influence on path loss, the results obtained with the six-wall model were on average 1.9 dB below the reference results. For this model, in the considered range of the receiving antenna distances, the difference was approximately −2 dB compared to the results obtained with the FDTD method. This model configuration was assumed to be the most suitable for simulating the body shadowing effect in the ray-based XGtd™ program.

The influence of the number of sidewalls used to model the body on path loss is significant. This was further investigated by the analysis of ray-paths identified by the program for two different body models and two locations of the receiver. In the abovementioned numerical experiment, the transmitter has a fixed position and the receiver is moved from the direct proximity of the body model to a 4 m distance. In the XGtd™ software, at each receiver location, contributions from arriving ray-paths are combined and evaluated to determine the predicted quantities of path loss. The calculation engine finds ray-paths that carry most of the energy of the electromagnetic wave from the transmitter to the receiver. The ray-path direction and properties for a particular location of the receiver depend on the geometry of the object. In the considered case, the number of prism sidewalls is critical because it influences the orientation of the top edges of the prism that are identified by the program as diffracting edges. The path loss at each point is then influenced by the diffraction coefficient of the object edges along the ray paths. Those depend on the angle between the ray and the edge of the object. This causes for several locations of the receiver the dynamic changes of path loss between the points for which the diffraction coefficient along the path changed significantly. 

**Figure 7:**
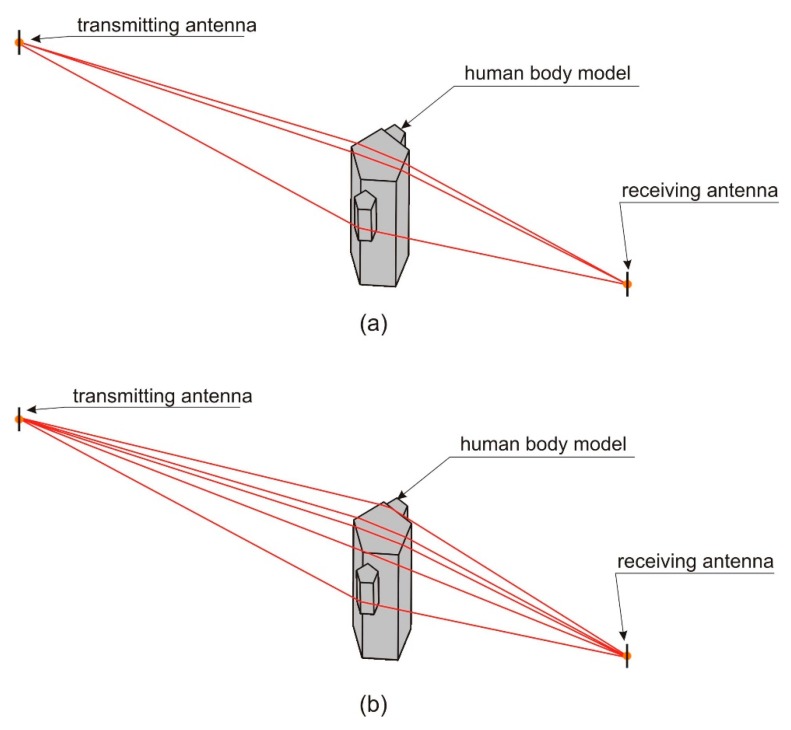
Ray-paths for human body models with five sidewalls: (**a**) For the receiver located at the distance, *b* = 1 m; (**b**) For the receiver located at the distance, *b* = 1.1 m.

To present this feature of XGtd™ software, the analysis of ray-paths identified by the program was made for two different body models and two locations of the receiver. In Figure 7, the ray-paths are presented for five walls’ model. For the receiver located at the distance, *b* = 1 m, there are three ray-paths identified by the program (Figure 7a) and in this case, the path loss was equal to 55 dB. The slight increase of the receiver distance to *b* = 1.1 m resulted with the five ray-paths identified by the program (Figure 7b) and the path loss decreased to 38 dB. In this case, not only the number of ray-paths is different, but also the diffraction coefficients change on the points where the path interacts with the object. This causes a rapid change in the path loss for small changes of the receiver to body distance. 

**Figure 8:**
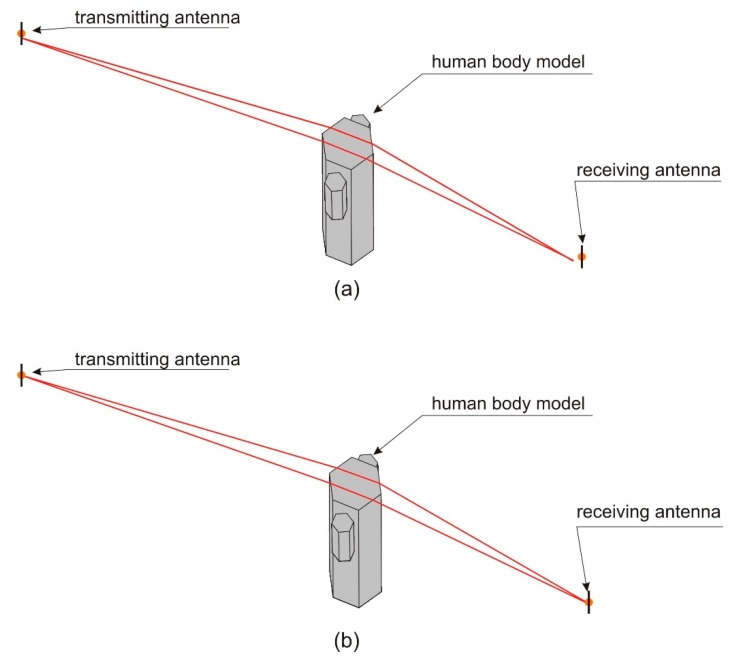
Ray-paths for human body models with six sidewalls: (**a**) For the receiver located at the distance, *b* = 1 m; (**b**) For the receiver located at the distance, *b* = 1.1 m.

A similar study was made for the model with six sidewalls. In Figure 8, the ray-paths for the human body model in this configuration are presented. The receiver was located at the distances, *b* = 1 (Figure 8a) and *b* = 1.1 m (Figure 8a). In both cases, there are two ray paths identified by the program. The path loss for both points (according to Figure 6) is approximately 56 dB. In the case of six sidewalls, there are no rapid changes of path loss, which makes it very similar to the reference model. This brief analysis shows that the number of sidewalls, and hence the number of edges and their spatial orientation, affect the results of path loss simulation in the XGtd™ program.

## 3. Results

Since many wireless sensor networks are used to monitor the physiological parameters of people, the analysis presented here of the influence of the human body shadowing effect on wireless sensor networks operating in the 2.4 GHz band was performed for a typical indoor environment. Analysis was first performed in the XGtd™ program using the human body model presented above. Experimental measurements were then performed in a similar indoor environment.

### 3.1. Analysis of Human Body Shadowing in an Indoor Environment Using XGtd™

The XGtd™ program is suitable for simulating the interaction of electromagnetic waves with large objects. It was therefore used to analyze the body shadowing effect in a wireless sensor network located in a typical hospital room. The multiple reflections from the walls in such a propagation environment made the path loss difficult to predict [42]. For this reason, computer models of path loss in indoor environments are particularly important for designers of wireless sensor networks. 

**Figure 9:**
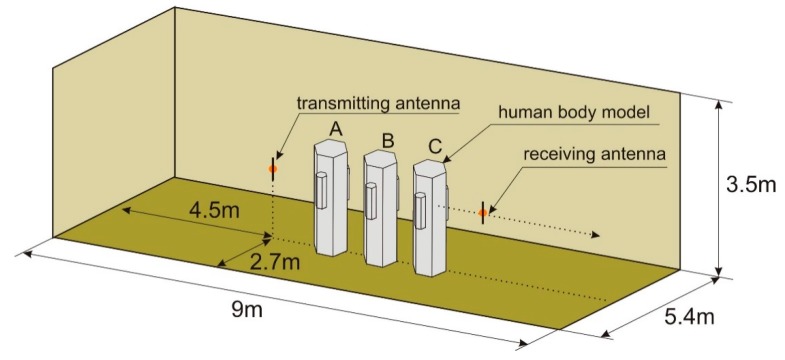
Geometry of the indoor scenario for simulating the shadowing effect in XGtd™ (picture not to scale).

The geometry of the indoor scenario for simulating the effect of shadowing is presented in Figure 9. The room is 9 m long and 5.4 m wide, and the ceiling height is 3.5 m. The walls, floor, and ceiling were modeled as being made of 35 cm thick concrete. The material properties of the concrete were taken from the material library available in XGtd™. The electric permittivity was equal to *ε_r_* = 15 and the conductivity was *σ* = 0.015 S/m. The transmitting dipole antenna that modeled the transmitting sensor was located in the middle of the room, 1.3 m above the floor. The receiving dipole antenna (of the receiving sensor) was placed at the same height, but its position was changed by approximately 1 m, from 2.5 m from the transmitting antenna to 3.5 m. 

The simulations were performed for a case with no human body located in the room and in the case of human bodies placed between the transmitting and receiving antennas. As shown in Figure 9, for the body shadowing case, three configurations were considered: One person placed in position “A”, two people in positions “A” and “B”, and three people in positions “A”, “B”, and “C”. The model with six side walls was used to simulate the human body.

**Figure 10:**
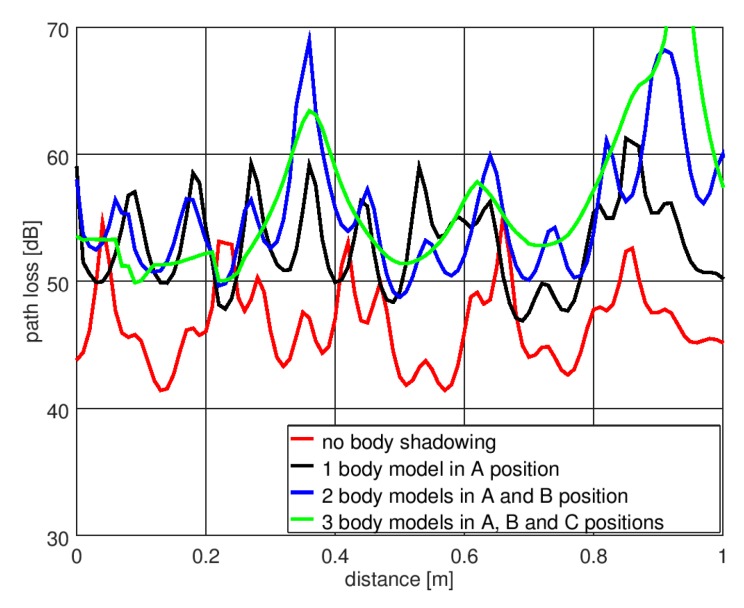
Results of path loss simulation for the indoor scenario in XGtd™.

Figure 10 presents the results of the path loss simulations in XGtd™ for the indoor scenario. Due to the multiple reflections from the walls, the path loss changes dynamically with changes in the position of the receiving antenna and the short-term fading is visible here. To compare the results obtained with and without the presence of human body models, the median path loss was calculated for displacements of the receiving antenna across a distance of 1 m. In the case of no human body between the antennas, the median path loss was 45 dB, the minimum value was 41 dB, and the maximum value was 55 dB. In the case of one person in position “A”, the median path loss increased to 54.5 dB, the minimum value was 46 dB, and the maximum value was 61 dB. For two people (in positions “A” and “B”), the median path loss was 55 dB, the minimum value was 49 dB, and the maximum value was 69 dB. For three people (in positions “A”, “B”, and “C”), the median path loss was 56 dB, the minimum value was 50 dB, and the maximum value was 79 dB. These results show the additional path loss that has to be included in the link budget analysis of wireless sensor networks operating inside buildings.

**Figure 11:**
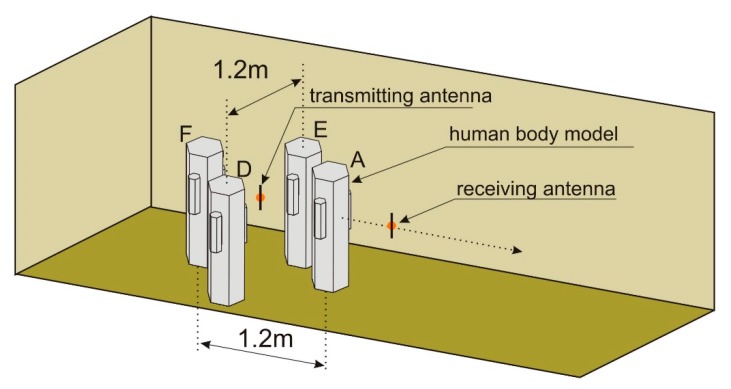
Geometry of the second scenario for simulating the shadowing effect in XGtd™.

The simulations were performed also for the second geometrical scenario, with human bodies placed around the transmitting antenna. As shown in Figure 11, for the body shadowing case, three spatial configurations were considered: Two persons placed in position “D” and “E”, three people in positions “A”, “D”, and “E”, and four people in positions “A”, “D”, “E”, and “F”. Apart from the location of people, all the geometrical parameters of the experiment were preserved from the previous case, as illustrated in Figure 9.

**Figure 12:**
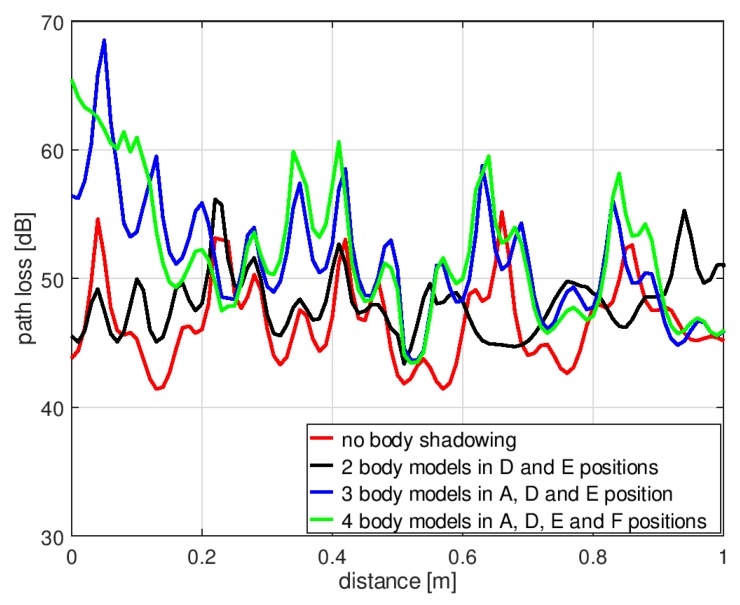
Results of path loss simulation for the second scenario in XGtd™.

In Figure 12, the results of path loss simulations obtained for the second scenario are presented. In the case of two persons in the “D” and “E” positions, there is no shadowing of the direct path between the transmitting and receiving antenna, but even in this case, the median path loss increased to 48 dB, the minimum value was 43 dB, and the maximum value was 56 dB. For three people (in positions “A”, “D”, and “E”), the median path loss was 51 dB, the minimum value was 44 dB, and the maximum value was 68 dB. In the case of four people (in positions “A”, “D”, “E”, and “F”), the median path loss was 52 dB, the minimum value was 43 dB, and the maximum value was 65 dB. These results show that the path loss caused by body shadowing depends on the spatial orientation of people in the indoor environment. For this reason, the use of numerical models of the human body in the discussed application is particularly important.

### 3.2. Measurements of Human Body Shadowing in the Indoor Environment

**Figure 13:**
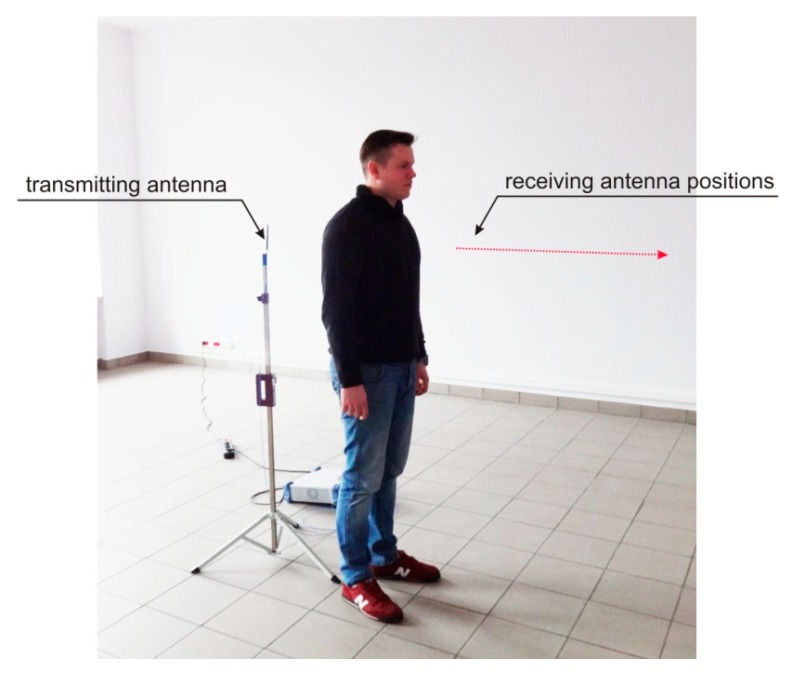
Setup for experimental measurements.

The results of computer simulations of the body shadowing effect performed with a simplified human body model in the XGtd™ program showed that the presence of a human body between the transmitting and receiving antenna can increase path loss significantly. To verify the results of the computer simulations, experimental measurements were performed in an indoor environment. The experiment was conducted in a room that had the same dimensions as the interior used for the simulations. A transmitter operating at 2.4 GHz was connected to a dipole antenna located in the middle of the room. The position of the receiving antenna was changed by approximately 1 m from 2.617 m to 3.67 m with 6 cm increments. Measurements were performed both for an empty room and with a human subject positioned between the transmitting and receiving antennas. The measurement setup is presented in Figure 13. 

**Figure 14:**
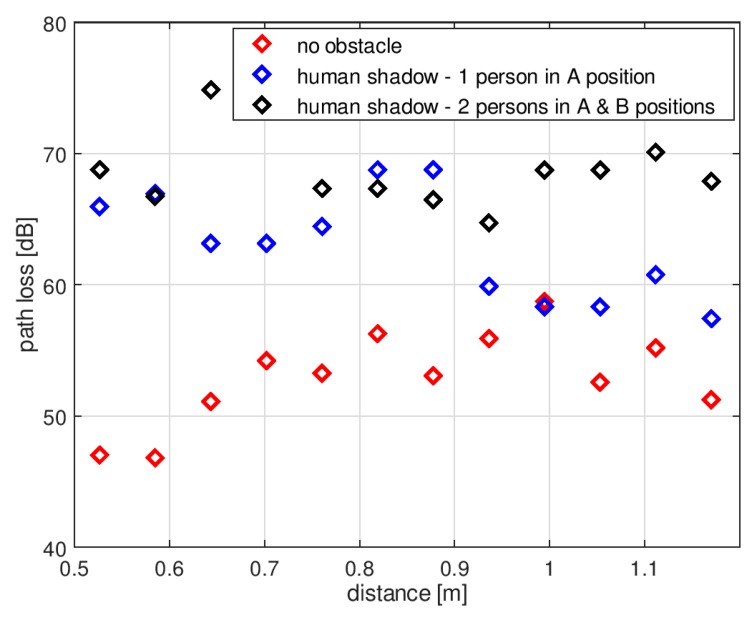
Results of path loss measurements in the indoor environment—first scenario.

Figure 14 presents the path loss measurements obtained for the first indoor scenario. Similar to the data obtained in simulations, the path loss changes dynamically with changing positions of the receiving antenna, which is the result of multipath propagation. For the purpose of comparison with the results of simulations obtained with and without human body models, the median, minimum, and maximum value was measured for the path loss with displacements of the receiving antenna over a distance of 1 m. The people were placed in the places showed in Figure 9. In the case of no human body between the antennas, the median path loss was 53 dB, the minimum value was 46 dB, and the maximum was 59 dB. In the case of one person in position “A”, the median path loss increased to 63 dB, the minimum value was 57 dB, and the maximum 68 dB. With two people in positions “A” and “B”, the median path loss was 68 dB, the minimum value was 65 dB, and the maximum was 75 dB. The presence of human bodies was therefore found to increase the path loss in the wireless link.

**Figure 15:**
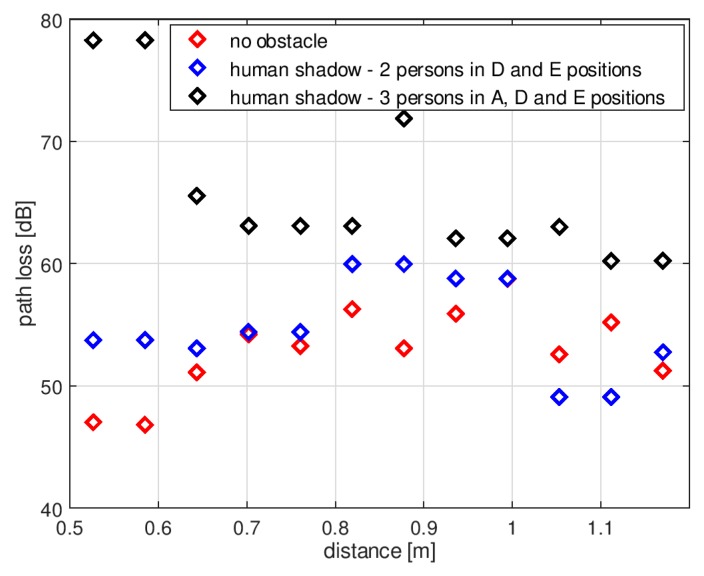
Results of path loss measurements in the indoor environment—second scenario.

Figure 15 presents the path loss measurements performed according to the second scenario presented in Figure 11. In the case of two persons in the “D” and “E” positions, the median path loss was 54 dB; that is 1 dB more compared to the result obtained with no obstacle. The minimum value was 49 dB and the maximum was 60 dB. With three people in positions “A”, “D”, and “E”, the median path loss was 63 dB, the minimum value was 60 dB, and the maximum was 78 dB. The presence of human bodies surrounding the transmitting antenna increased the path loss also in this geometrical case.

## 4. Discussion

The results presented above were obtained from both computer simulations and measurements of the body shadowing effect in wireless sensor networks for the very popular 2.4 GHz band. The computer simulations performed with the full wave FDTD method showed that the presence of a human body can increase path loss significantly. When a human body is close to the wireless sensor, path loss may increase (from 10 dB up to 20 dB) and could exceed the fading margin of the link. 

Computer simulations of path loss in wireless sensor networks using FDTD-based software are limited, however, to a relatively small range of distances. To model wireless sensors located 7 m from each other with a human body in between, 9.8 GB of memory on a computer equipped with GPU cards was required. The time required with this setup for the simulation of a single position of the receiving antenna was up to 85 min. This shows the limitation of FDTD-based software for simulating the body shadowing effect on wireless sensor networks that operate inside buildings. For such networks, it is necessary to model much larger areas and also to include the walls and other obstacles. For this, FDTD-based programs would require a computer with far more than 12 GB of memory, while the time needed for the simulations would be impractically high, especially if the wireless sensor network design demanded numerous simulations. 

The body shadowing effect can be successfully modeled using ray-based programs that apply the UTD method for simulating the interactions of electromagnetic waves with human bodies. In the present study, Remcom XGtd™ software was used with a human body model proposed for the purposes of the analysis. This required simplification of the human body shape, which was constructed using prisms with varying numbers of sidewalls. The smallest differences between the simulations of path loss made using FDTD and UTD methods were obtained using a model with six side wall prisms. When the receivers were located close to the human body, where the body shadowing effect has the strongest influence on path loss, the results obtained with the six-wall model were on average 1.9 dB below the reference results obtained with FDTD. With this model, across the whole considered range of receiving antenna distances, the difference between the results obtained using UTD and FDTD was approximately −2 dB. This difference is sufficiently small to allow the model to be used to estimate the fade margin that should be assumed for successful transmission in wireless sensor networks operating in the presence of human bodies. 

The proposed model of a human body was used to analyze the influence of body shadowing on a wireless sensor network operating in an indoor environment. Due to multiple reflections from the walls, the path loss changed dynamically when the receiving antenna was moved. When there was no human body between the antennas, the median path loss calculated for different positions of the receiving antenna across a distance of 1 m was 45 dB. In the case of one person shadowing the transmitter, the median path loss increased by 9.5 dB. With two people, the median path loss increased by 10 dB. With three people, the median path loss increased by 11 dB. This shows that the presence of human bodies in the considered geometry of the experiment caused additional path loss of approximately 10 dB. For wireless sensor networks that operate with low power transmitters, this value should be considered in the link budget analysis for building interiors.

The path loss simulations in the second scenario were performed for the case where there are two persons in the proximity of the transmitting antenna, but there is no shadowing of the direct path between the transmitting and receiving antenna. It was shown that even in this case, the median path loss increased to 48 dB, the minimum value was 43 dB, and the maximum value was 56 dB. These results show that in the indoor environment where multipath propagation occurs, the path loss depends on the spatial orientation of people. This effect can be studied with the proposed model of the human body. 

The body shadowing effect was also observed in experimental measurements conducted in a typical building with concrete walls. In this case, the additional path loss introduced by the presence of a person was equal to 10 dB, while with two people standing close to each other and fading the transmitter path loss increased by approx. 15 dB. These measurement results correspond well with the results of simulations using ray-based software and the proposed human body model based on six-wall prisms.

The observed differences between absolute values of path loss obtained with the measurements and the simulations may be caused by the simplification of the building structure that was made in the XGtd™ program. The room was modelled with four walls, a floor, and ceiling made of concrete with the same thickness and material properties. In a real building, the structure of the walls is different than the structure of the ceiling and the floor. With those simplifications, it was possible to analyze the influence of the human body on the path loss by the relative change of this parameter. 

## 5. Conclusions

The research presented in this paper shows that the human body shadowing effect influences the performance of wireless sensor networks operating in the 2.4 GHz band. This effect introduces additional path loss that should be considered by designers of networks that utilize low power miniature sensors with limited transmit power and a small fade margin. The shadowing effect can reduce the operational range of networks, especially in the case of indoor networks where people may be present and the relative positions of the antennas and obstacles may vary.

Computer models that use the FDTD method are suitable only for analyzing the body shadowing effect on networks that operate across short distances. The numerical cost of the simulations makes the FDTD method impractical for analyses of networks that cover entire buildings or groups of buildings. To simulate the body shadowing effect on wireless sensor networks that operate inside buildings, a ray-based computer program can instead be used. This paper proposed a simplified human body model elaborated in the Remcom XGtd™ program for simulations of the body showing effect in indoor scenarios, where multiple reflections should be considered. The model can be used to estimate the fade margin that should be assumed for successful transmission across a wireless sensor network operating in the presence of human bodies. 

The proposed model of the human body was used for the analysis of the body-shadowing effect in the indoor environment with the XGtd™ program. The results were obtained for two scenarios with a different number of body models that were placed in a few orientations towards the transmitting and receiving antennas. The simulations showed that the presence of human bodies in the proximity of the transmitter causes additional path loss that, in the case of wireless sensor networks that operate with low power transmitters, should be considered in the link budget analysis for building interiors. It was shown that the path loss in the 2.4 GHz band in the indoor environment depends strongly on the number of people as well as on their spatial orientation in the propagation environment. This effect can be effectively studied with the proposed model of the human body. 

The simulations of path loss were followed by measurements that also identified the additional path loss introduced by the presence of a person. These measurement results correspond well with the results of simulations using ray-based software and the proposed human body model based on six-wall prisms. The small differences observed between absolute values of path loss obtained with the measurements and the simulations may be caused by the simplification of the building structure that was modeled in the XGtd™ program. 

Further studies are planned to investigate the possibility of reconfiguring the proposed model for use in additional frequency bands.

## Figures and Tables

**Table 1:** Differences between median values for path loss obtained using UTD and FDTD with different numbers of sidewalls approximating the main body cylinder across three distance ranges.

Number of Sidewalls	Difference between Median Path Loss [dB] Obtained with UTD and FDTD Across Particular Distance Ranges		
	0.5 m–1 m	1 m–2 m	2 m–4 m
5	−2.7	−12.6	−8.3
6	−1.9	−1.7	−2.3
7	−11.6	−9.2	−4.1
8	−3.2	−13	−13.7
